# Relationship between neck kinematics and neck dissability index. An approach based on functional regression

**DOI:** 10.1038/s41598-023-50562-x

**Published:** 2024-01-02

**Authors:** Elisa Aragón-Basanta, William Venegas, Guillermo Ayala, Alvaro Page, Pilar Serra-Añó

**Affiliations:** 1 Camino de Vera s/n, Instituto Universitario de Ingeniería Mecánica y Biomecánica, Universitat Politècnica de València, 46022 Valencia, Spain; 2 Facultad de Ingeniería Mecánica, Escuela Politécnica Nacional, PO-Box 17-01-2759, Quito, Ecuador; 3 Avda Vicent Andrés Estellés 1, Departament of Statistics and Operation Research, Universitat de València, 46100 Burjasot, Spain; 4 Gascó Oliag 5, Departament of Physiotherapy, Universitat de València, 46010 Valencia, Spain

**Keywords:** Biomedical engineering, Mechanical engineering

## Abstract

Numerous studies use numerical variables of neck movement to predict the level of severity of a pathology. However, the correlation between these numerical variables and disability levels is low, less than 0.4 in the best cases, even less in subjects with nonspecific neck pain. This work aims to use Functional Data Analysis (FDA), in particular scalar-on-function regression, to predict the Neck Disability Index (NDI) of subjects with nonspecific neck pain using the complete movement as predictors. Several functional regression models have been implemented, doubling the multiple correlation coefficient obtained when only scalar predictors are used. The best predictive model considers the angular velocity curves as a predictor, obtaining a multiple correlation coefficient of 0.64. In addition, functional models facilitate the interpretation of the relationship between the kinematic curves and the NDI since they allow identifying which parts of the curves most influence the differences in the predicted variable. In this case, the movement’s braking phases contribute to a greater or lesser NDI. So, it is concluded that functional regression models have greater predictive capacity than usual ones by considering practically all the information in the curve while allowing a physical interpretation of the results.

## Introduction

Neck pain is a complex condition that can be caused by multiple factors, and it is a significant issue in modern society. It is the second most common musculoskeletal pain condition worldwide, and its prevalence has increased significantly over the last three decades^1^. The economic burden of neck pain is remarkable and includes treatment costs, reduced productivity, and job-related problems^2,3^. Therefore, finding a cost-effective diagnostic method is important to better characterize the consequences of pain and manage the patients who suffer it.

Neck kinematics analysis is helpful in assessing the severity of neck injuries since pain and functional losses are related to mobility impairments. Traditionally, mobility has been quantified by the cervical range of motion (CROM), the reduction of which is associated with numerous pathologies^4^.

However, CROM provides incomplete information about kinematics and fails to explain how movement occurs within the joint range. To address this limitation, other studies have focused on measuring continuous motions, capturing angular positions, speeds, accelerations and other kinematic parameters related to mobility and motor coordination. Generally, reduced range, slower speed, less smoothness, and decreased reproducibility of movements are often associated with pain and disability. There is a wealth of information available on kinematic studies, which has been recently reviewed^5^.

Despite their potential interest, the variables extracted from kinematic studies, such as CROM, maximum speeds, accelerations, and smoothness of movement, exhibit high variability. While differences between the means of these variables for healthy individuals and those with pathology have been observed, these differences are not as pronounced at the individual level. Consequently, establishing cut-off values becomes challenging, limiting the clinical utility of these techniques^5^.

The relationship between the kinematic variables and the assessment obtained with other widely used and documented clinical assessment tools, such as self-reported disability questionnaires, is generally weak. These tools, including questionnaires such as the Neck Disability Index^6^, the Visual Analogue Scale (VAS)^7^, the TAMPA scale for kinesophobia^8^, among others, have good reliability values and are widely used in the clinical setting^9,10^.

Some studies have been carried out that analyze the relationship between scale scores and kinematic variables. Most studies use CROM as a reference variable using other measurement methods such as inclinometers^11–13^, goniometers^14–16^ or radiographs^17,18^. Although most studies report a decrease in CROM as pain score or disability index increases, correlations are low, with absolute values between 0.1 and 0.4 in most studies^9^. However, it is difficult to compare the studies because they use different measurement techniques and are applied to patients with varying types of injuries and levels of severity.

Less numerous are those that analyze the relationship between the kinematic variables obtained from continuous movements and the questionnaires^19–24^. These studies offer scattered results due to the variability in the type of tests carried out and the differences in the pathologies analyzed. However, there is evidence that as pain or disability increases, movements are slower, and the correlations are not too high either, generally less than 0.4, except in some studies with a small number of samples and subjects with acute pain or high levels of disability.

It is therefore worth considering the reason why variables associated with the movement have such low relationships with disability indices whose reliability and clinical utility have been proven^9^.

One possible cause is the dispersion in the individual response in kinematic tests, which involve voluntary movements with notable variability, leading to high values of the minimum detectable difference^25^. This dispersion is even more significant in the case of variables such as speeds and accelerations^26^. On the other hand, the reliability of the ROM is not better than that reported for scales such as the NDI, whose Intraclass Correlation Coefficient (ICC) values exceed the value of 0.9 in many pathologies^27,28^. These values are never reached for variables such as speeds or accelerations in kinematic tests^26,29^.

In addition to these causes, associated with the limited reproducibility of kinematic techniques, it is also worth asking what information is extracted from the continuous movement records and what use is made of said information. Most studies reduce the motion curve information to just a few parameters, such as maximum or minimum speed, acceleration, or a mean value. Using the ranges or the maximum values is an easy way to obtain numerical values from a curve. Still, it supposes a loss of important information since a few values reduce the kinematic information of each curve. Furthermore, such variables are chosen in a possibly arbitrary manner, generally at points that are easy to identify.

The movement of a joint is described by the curves of positions, speeds, and accelerations that, from the mathematical point of view, can be described and treated as functional variables within the framework of the so-called Functional Data Analysis (FDA). This branch of Statistics extends and generalizes the classical methods of numerical variables to functional variables^30,31^. The use of FDA techniques has essential advantages over the classical description of the movement since it maintains all the information contained in the curves (and in their derivatives) without reducing them to a set of numerical values (maxima, minima, event durations) that do not represent the relationships associated with motor coordination and movement dynamics.

There have been numerous studies in the field of FDA-based biomechanics^32,33^ that include generalizations of classical techniques to describe the variability of curves using functional principal component analysis^34,35^, analyze the differences associated factors using functional ANOVA^36,37^, the shape variation of curves associated with numerical variables through functional regression^38,39^ and curve-based classification techniques^40,41^.

We have not found any studies that examine the correlation between movement curves and clinical indices that represent pain or disability. In the clinical setting, there are multiple indices to evaluate pain or disability caused by pain. The NDI is the most commonly used tool to assess self-rated disability in patients with neck pain. This instrument encompasses various clinical domains, such as pain intensity and the ability to perform basic and instrumental daily activities. To explore the relationship between movement curves and this neck clinical index, a variant of functional regression with numerical response (for example, the NDI) and functional predictors (motion curves) i.e. scalar-on-function regression could be used. The general mathematical approach was proposed by Ramsay and Silverman^30^. However, there are a few medical applications of this type of regression^42^. As far as we know, it has never been used to interpret the relationship between motion curves and disability indices.

The subject of this paper is to use functional regression to identify the functional relationship between movement curves and NDI scores in a sample of subjects with non-specific neck pain. It will be verified how the correlation between the kinematic variables and the NDI is improved using all the information on the movement curves. It also provides a way of interpreting the contribution of each part of the movement to the increment or decrement of the given response i.e., the index considered.

## Materials and methods

### Materials

The data on neck movement kinematics correspond to a previous study designed to analyze the effect of a manipulation session in patients with non-specific neck pain^43^. In said study, only conventional numerical variables (ranges of movement and velocity) were analyzed, although complete movement functional data were recorded, which are the ones analyzed in the present study.

Twenty-eight subjects participated in the study. Participants eligible for inclusion met the following criteria: a primary complaint of neck pain characterized by a non-traumatic onset and a mechanical nature (i.e., pain exacerbated by mechanical factors and alleviated in specific positions). Additionally, identifiable symptoms were produced through local clinical provocation tests. Exclusion criteria for the participants encompassed a history of neck surgery, the presence of radiological findings such as vertebral fractures or tumors, radiating neck pain or pain coupled with vertigo, diagnosed psychological disorders, and red flags such as night pain, severe muscle spasm, and involuntary weight loss. Symptom mismatch, defined as unexplained symptoms outside the clinical context, was also considered in the exclusion criteria. Measures of movement and functional status were obtained for each subject before and after treatment, giving a total set of 56 observations. Both measures were used for each subject due to significant differences in NDI values before and after treatment and because they were obtained in different sessions. For this sample size, a power of 0.8 is obtained (with $\alpha =0.05$) for correlation coefficients greater than $r= 0.22$^44^.

All the participants signed an informed consent, and the study protocols were approved by the Ethics Committee of the University of Valencia (H1450106985729). All procedures were performed in accordance with the latest revision of the Declaration of Helsinki.

The kinematic study performed neck flexion-extension tests according to the protocol described in Venegas et al.^26^, whose general lines are described below. The subject sat in a chair designed for this purpose, which allows fixing the position of the trunk and legs so that only neck and head movement is allowed. Said movement was recorded using a video-photogrammetry system (Kinescan-IBV) using eight reflective markers on a headband attached to the subject’s head. As a starting reference position, the participants were asked to look at their eyes in a small mirror ($3\times 8$ cm.) placed 2.5 m. in front of them at eye level. An additional calibration measurement was performed to define an anatomical reference system in this position. This system was defined by an additional set of 5 markers located on the left and right tragus of the ear, on the nasal bone, and the left and right infra-orbital bones; the latter three markers were mounted on a spectacle frame. After calibration, the anatomical markers were removed.

In each measurement session (before and after treatment), subjects had to perform cyclical and continuous neck flexion-extension movement at the maximum speed they considered comfortable.

From the coordinates of the markers, the angles from the reference position and the velocities were calculated using the calculation process described in Page et al.^45^. A continuous record was obtained in each session, divided into complete extension-flexion cycles (7 per subject), of which the first and last were discarded, i.e., we have five cycles per individual. Subsequently, the time scale was normalized linearly so that all movements were represented as percentages of the cycle duration. Later, the functional mean of the five position and angular velocity curves was obtained and used as independent functional variables in this work. In addition, the ranges of motion (ROM) were calculated as the difference between the maximum and minimum angles and the corresponding ranges of angular velocity, which are the predictors for the regression model.

The Spanish version of the Neck Disability Index (NDI) was the dependent variable^46^. This questionnaire assesses the ability of users to perform functional tasks of daily life. The NDI comprises ten questions categorized into the following domains: Pain Intensity, Personal Care, Lifting, Reading, Headaches, Concentration, Work, Driving, Sleeping, and Recreation. Within each question, respondents choose from six possible answers, with scores ranging from 0 (indicating no disability) to 5 (reflecting complete disability). The individual scores for each section are then summed up. The overall scoring is presented on a scale of 0 to 50, where 0 represents the optimal score, indicating no disability, and 50 signifies the poorest score, indicating the highest level of disability. This questionnaire was filled before starting each measurement session (before and after treatment) so that a measurement of the numerical dependent variable is available for each observation of the functional independent variables.

### Methods

#### Model

Our goal is to model the conditional distribution of a scalar given functions and scalars. A common practice just mentioned is to describe each functional predictor with some numerical descriptors such that functions and scalars are used together as scalars, resulting in loss of information. This practice allows us to use linear models and generalized linear models to model the distribution of the response conditional to given numerical predictors. This paper tries to widen the point of view and show how to use functional predictors on its own. Let us show the way from the simple regression model to functional regression.

If we have a unique scalar predictor, $x_i$, and a scalar response, $y_i$ then our data set consists of $(x_i, y_i)$ with $i=1,\ldots ,n$ where a given $x_i$ could correspond to different responses. The interest is to model the probability distribution of the random response $Y_i$ given the predictor $x_i$, and the simplest regression model is

1 $$\begin{aligned} Y_i = \beta _0 + \beta _1 x_i + \epsilon _i, \end{aligned}$$

where the random errors $\epsilon _i$’s are assumed  independent and normally distributed with null mean and common variance $\sigma ^2$ i.e., $\epsilon _i$ are i.i.d. (independent and identically distributed) with $\epsilon _i \sim N(0,\sigma ^2)$. If several scalar predictors, $\varvec{x}_i \in {\mathbb {R}}^p$, are used, then the multiple regression model assumes that $Y_i$ given $\varvec{x}_i$ is given by

2 $$\begin{aligned} Y_i = \sum _{j=1}^p x_{ij} \beta _j + \epsilon _i = \varvec{x}_i^T \varvec{\beta }+ \epsilon _i, \end{aligned}$$

where $\varvec{x}_i^T$ is the transpose of the column vector $\varvec{x}_i = (x_{i1},\ldots ,x_{ip})$ and $\varvec{\beta }= (\beta _1, \ldots , \beta _p)$ with $\epsilon _i$’s are i.i.d. with $\epsilon _i \sim N(0,\sigma ^2)$ i.e. it is assumed that $Y_i \sim N(\varvec{x}_i^T \varvec{\beta },\sigma ^2)$.

If a functional predictor, $x_i$, is available and a random scalar response, $Y_i$, then the natural extension of the simple regression model is

3 $$\begin{aligned} Y_i = \beta _0 + \int \beta _1(t) x_i(t) dt + \epsilon _i, \end{aligned}$$

with the random errors $\epsilon _i$’s i.i.d. with $\epsilon _i \sim N(0,\sigma ^2)$. It is assumed that $x \in L^2$ i.e., $\int _T x^2(t) dt < \infty$ i.e. the square of the function is integrable. Let us remember that for two functions defined as a domain *T*, $f,g \in L^2(T)$, the inner product is defined as $<f,g> = \int _T f(t)g(t)dt$. The model (3) can be rewritten as

4 $$\begin{aligned} Y_i = \beta _0 + <x_i,\beta _1> + \epsilon _i\ \text {for}\ i=1, \ldots , n. \end{aligned}$$

A *numerical* coefficient corresponding to a scalar predictor is replaced by a *functional* coefficient corresponding with a *functional* predictor in the simple regression model given in (1). The functions $x_i$ and $\beta _1$ are expressed in (possibly) different basis: $x_i(t) = \sum _{r=1}^{K_{x_1}} c_{i r} \psi _r(t)$ and $\beta _1(t) = \sum _{s=1}^{K_{\beta }} b_s \theta _s(t)$ in such a way that

5 $$\begin{aligned}{} & {} \int \beta _1(t) x_i(t) dt = \int _T \sum _{r=1}^{K_{\beta _1}} b_r \theta _r(t) \sum _{k_2=1}^{K_x} c_{i k_2} \psi _{k_2}(t) dt = \nonumber \\{} & {} \quad \sum _{k_1=1}^{K_{\beta _1}} \sum _{k_2=1}^{K_x} b_{k_1} c_{i k_2} \int _T \theta _{k_1} (t) \psi _{k_2}(t) dt = \sum _{k_1=1}^{K_{\beta _1}} \sum _{k_2=1}^{K_x} c_{i k_2} J_{k_1 k_2} b_{k_1} = \sum _{k_1=1}^{K_{\beta _1}} v_{k_1} b_{k_1}, \end{aligned}$$

where $v_{k_1} = \sum _{k_2=1}^{K_x} c_{i k_2} J_{k_1 k_2}$ is known. The multiple regression model would be

6 $$\begin{aligned} Y_i = \beta _0 + \sum _{j=1}^{K_{\beta _1}} v_j b_j + \epsilon _i, \end{aligned}$$

where the $b_j$’s are the coefficients. The coefficients $c_{ik}$ are estimated using least squares. The basis chosen and the number of functions of this basis are fundamentals in the shape of the coefficient function.

If several functional predictors $x_i^{(s)}$ with $s = 1, \ldots , S$ are considered besides scalar predictors $\varvec{u}_i \in {\mathbb {R}}^q$ then the functional regression model can be formulated as

7 $$\begin{aligned} Y_i = \beta _0 + \sum _{s=1}^S <x_i^{(s)},\beta _s> + \sum _{r=1}^q \beta _r^{(\varvec{u})} u_{ir} + \epsilon _i. \end{aligned}$$

It can be chosen different basis functions for the different coefficient functions $\beta _s$. Additionally, note that the functional predictors have to be expressed in its own basis.

#### Family and number of basis functions

There are two procedures to estimate the coefficient function $\beta _s$ (3). Both procedures use basis expansions of $\beta _s$ with the following difference: The first one uses a low-dimensional basis for $\beta _s$ while the second uses a combination of a high-dimensional basis with a roughness penalty.

For this paper, the first option is preferred due to the fact of a better interpretation of the regression coefficient function and the good results obtained, which will be explained later. A basis of functions for the coefficient function is required. There are several possibilities: B-splines, Fourier, exponential, $\ldots$ For periodic data, such as the flexion-extension movement of the neck, the Fourier function basis is usually used. The B-splines function basis, also widely used, is more used for non-periodic functional data^30^. Fourier basis has been chosen: $\{1, \sin (\omega t), \cos (\omega t), \sin (2\omega t), \cos (2\omega t), \dots , \sin (k\omega t), \cos (k\omega t), \dots \}$.

Then, it is required to establish a criterion to choose the optimal number of basis functions providing a good data approximation and a robust model. Obviously, more basis functions will correspond with a higher multiple correlation coefficient but a less robust model. In addition, the coefficient function has a more complicated interpretation with respect to the movement analyzed. We will take into account three different criteria in order to choose the basis dimension. The first criterion is to achieve a good multiple correlation (denoted *r*). Secondly, it has been tested if all coefficients can be considered simultaneously null, i.e., the nested model with all predictors and the model with just the constant are compared. The usual *F* statistic or the corresponding *p*-value can be used. Finally, the third criterion is the widely used Akaike Information Criterion (AIC)^47^, defined as minus twice the maximum loglikelihood of the model plus twice the number of parameters of the model.

A good number of basis functions will correspond with higher values of the correlation coefficient *r*, lower *AIC* and a reasonable value of *F* statistic. All calculations have been carried out with the functional regression model of velocity, which is the simplest one.

#### Statistical analysis

To test the hypotheses, the following regression models have been fitted. In all of them, the NDI is the response and the different predictors are shown in Table 1.

**Table 1:** Four scalar-on-scalar regression models using the range of angle (RoM) and the angular velocity (RoV). Two scalar-on-function regression models consider the angle $\varphi (t)$ and the angular velocity $\omega (t)$. Two scalar on scalar and function regression models. The models are named using the R formula notation extended to include functional predictors. When there is a functional predictor then the corresponding function is indicated. For example, $ndi \sim RoM$ corresponds to $ndi_i=\beta _0+\beta _1 RoM_i+\epsilon _i$. The notation of $ndi \sim RoM + RoV + RoM:RoV$ in the last non-functional model incorporates the interaction between both predictors.

Predictor type	Model
Scalar	$ndi \sim RoM$
	$$ndi \sim RoV$$
	$$ndi \sim RoM + RoV$$
	$$ndi \sim RoM + RoV + RoM:RoV$$
Function	$ndi \sim \varphi (t)$
	$$ndi \sim \omega (t)$$
Scalar and function	$ndi \sim \omega (t) + RoV$
	$$ndi \sim \omega (t) + RoV + RoM$$

The position and velocity (numerical or functional) have been chosen because they are the most used in clinical practice. In the case of the models with scalar and functional predictors, we have only considered the angular velocity as a functional variable because it offers better fits than the angle as will be verified later.

In the first analysis, each model has been evaluated using the multiple correlation coefficient, *r*, the determination coefficient $R^2$, the statistic *F* testing all coefficients except the constant as null and the *p*-value corresponding to this *F* statistic. The same basis has been used in all functional models selected according to the above mentioned criteria.

On the other hand, a nested model has been fitted to evaluate the improvement of functional models compared to non-functional ones. In the first analysis, the improvement of the NDI vs. $\omega (t)$ model is analyzed when the two scalar variables *RoM* and *RoV* are added. Then, we study the improvement of adding the functional variable to the two scalar variables. A hypothesis test is carried out where the null hypothesis $H_0$ is that both models are not different i.e. all coefficients in the more complex model and not in the simpler one are null. In this way, if the *p*-value is high, there is no evidence to reject the null hypothesis, so the variables of the more complex model do not improve the simpler model. On the other hand, if the *p*-value is small, there is evidence to reject the null hypothesis, then the coefficients are not null, and the variables added to the model $M_0$ improve the results of the simplest model.

All analyses have been carried out with the R packages fda^48^ and fda.usc^49^.

## Results

### Participants and kinematic data

Table 2 shows some numerical descriptions of the subjects participating in the study. There are 28 subjects (18 women and 10 men) whose neck movement and NDI have been measured before and after treatment. Significant differences were found in the NDI means before and after treatment (*p*-value <0.001 obtained by a paired *t*-test). Therefore, there are 56 different observations, of which only 55 have been considered in this paper because one of the observations had errors in the measurements of the movement curves.

**Table 2:** Some descriptions of the subjects studied: the sample size for women and men, the mean and standard deviation of age, weight, height, and NDI before and after treatment. There are significant differences in the NDI before and after the treatment (*p*-value<0.001).

VARIABLE	WOMEN (N=18)	MEN (N=10)	TOTAL
	Mean (sd)	Mean (sd)	Mean (sd)
Age	35.8 (12.4)	33.3 (13.4)	34.9 (12.6)
Weight	64.9 (10.1)	83.6 (11.9)	71.6 (14.0)
Height	162.6 (6.8)	178.8 (7.35)	168.4 (10.5)
NDI (before)	13.6 (3.7)	9.7 (4.1)	12.2 (4.2)
NDI (after)	9.4 (6.2)	7.0 (3.5)	8.6 (5.5)

**Figure 1:**
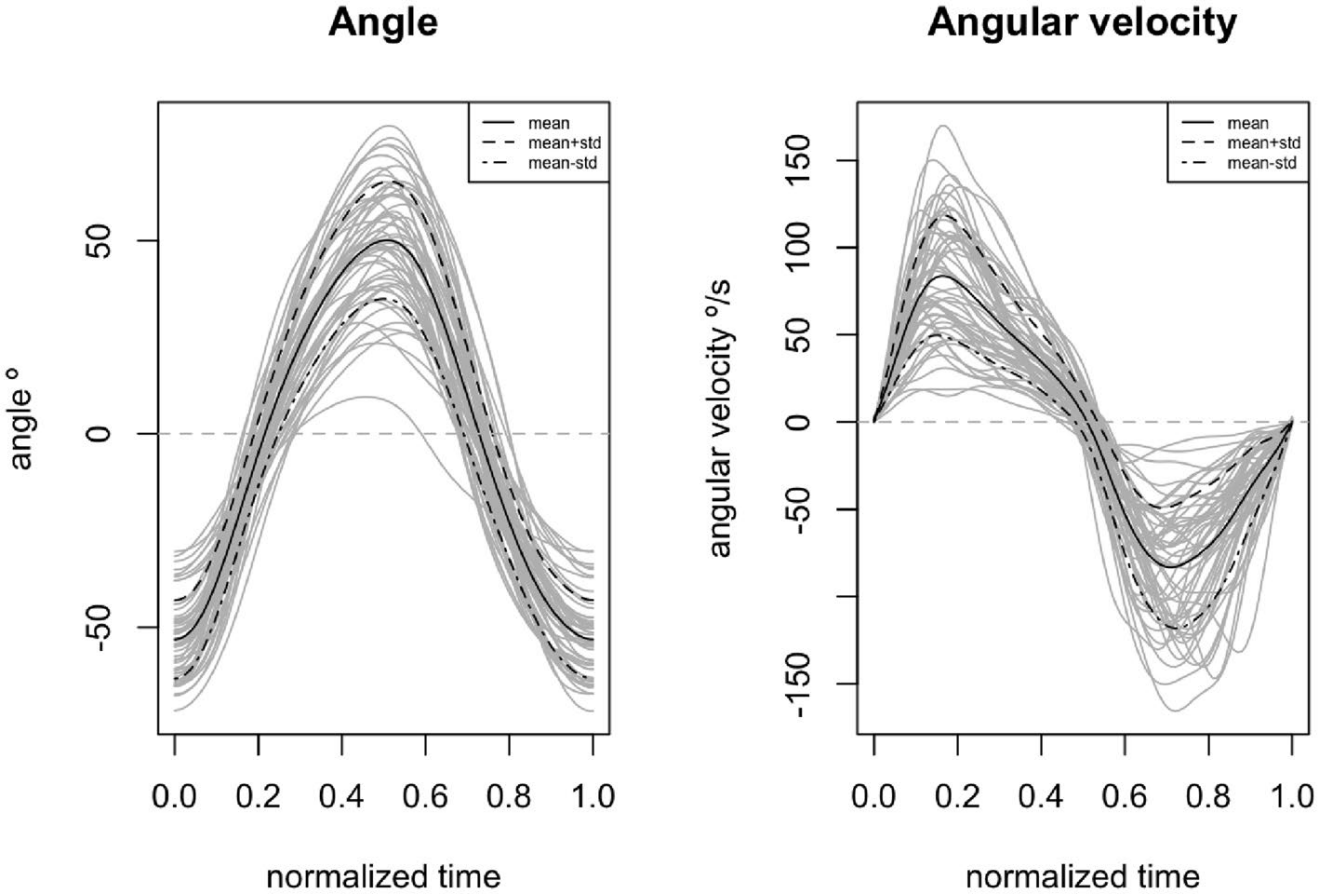
Curves of angle and angular velocity. **Left**, angle, **right**, angular velocity. The grey lines are the curves for each subject, black line is the mean functional curve, the dashed black line is the functional mean plus one standard deviation and finally, the dot-dashed black line is the functional mean minus one standard deviation.

The curves of angle and angular velocity are shown in Fig. 1, where the curves of all observations (N=55) are represented, as well as the mean curve and the curves of the mean plus and minus one standard deviation, respectively.

### Number of functions

Table 3 shows the results corresponding to the selection criteria for the number of functions of the Fourier basis: the multiple correlation coefficient *r*, the statistic *F* and *AIC*. Note that there is an odd number of functions because the Fourier basis has pairs of sine and cosine functions. Regarding the results in Table 3, the number of functions must be chosen considering the best criterion values as a whole since the improvement of some implies a worsening of others. Thus, although five functions provide the highest *F* value, they give the lowest multiple correlation value with little difference in the value of AIC if seven or nine functions are considered. Therefore, we choose nine functions as a compromise solution with a high multiple correlation value (0.64), an AIC similar to the one obtained with fewer functions, and a good F, not the highest value but not the lowest. In conclusion, we will consider nine functions to define our Fourier basis, i.e., the constant and four pairs of sines and cosines.

**Table 3:** Observed *r*, Akaike Information Criterion *AIC*, statistic *F* and *p*-value for different number of basis functions of $\beta _s$ using as predictor the angular velocity.

Number of basis	*r*	*AIC*	*F*	*p* value
5	0.58	160.47	4.88	0.001
7	0.60	161.62	3.88	0.002
9	0.64	161.68	3.48	0.003
11	0.64	165.43	2.75	0.009

### Model comparison

Table 4 shows different measures of goodness for the fitted models: non-functional, functional, and scalar and functional. The non-functional models present poor multiple correlation values, $r<0.352$ in the most complete model. The fit with RoV is much better than when using RoM ($r= 0.298$ vs. $r=0.017$), a model whose *p*-value is non-significant ($p=0.902$). Adding variables and interactions somewhat improves the correlation but at the cost of lowering the *F* statistic and the *p*-value.

The functional models improve the correlation with the NDI and present significant *F* values. Thus, the model that adjusts the angles $\varphi (t)$ shows a multiple correlation $r=0.563$, compared to $r=0.017$ when the RoM is used. The improvement is also notable in the case of angular velocity ($r=0.640$ in the functional case, compared to $r=0.298$ in the RoV).

The angular velocity $\omega (t)$ model is better than the one based on the angle $\varphi (t)$ in all indicators. It presents a higher multiple correlation (0.64 versus 0.563), a higher *F* value (3.475 versus 0.317), and a lower *p*-value (0.002 versus 0.301). In addition to presenting worse fits, it should be noted that the function $\varphi (t)$ is a geometric variable that depends on the reference taken as the neutral position. On the contrary, $\omega (t)$ is a physical variable that takes values associated with the movement that do not depend on any geometric reference. Therefore, $\omega (t)$ has been used as the only functional predictor in the functional plus scalars models with the other scalar predictors.

Such functional plus scalars models do not substantially improve the simpler functional model based on angular velocity. Thus, the model that adds RoV presents the same *r* but lower *F* values (3.060 vs 3.475) and higher *p*-values (0.005 vs. 0.002). The model that uses $\omega (t)$, RoV, and RoM barely increases the multiple correlation coefficient by a few thousandths (0.643 vs. 0.640), with a notable reduction of *F* (2.755 vs. 3.475) and an increase in the *p*-value (0.009 vs. 0.002).

To test the hypothesis that the functional model based on angular velocity is better than the scalar models, the model that includes the functional predictor, $\omega (t)$, and the two scalars RoM and RoV have been analyzed. This model, $M_1$, has been compared with two simple nested models (Table 5). The first model, $M_{01}$, is the simple functional model with $\omega (t)$ as the only predictor. The second, $M_{02}$, is the model with the two scalar predictors.

The results in Table 5 show that the improvement of the model by incorporating the two scalar variables is not significant (*p*-value= 0.884), which implies that it cannot be rejected that the coefficients corresponding to scalar predictors are null. Likewise, the improvement in the purely scalar model when adding the functional predictor is significant ($p=0.028$).

**Table 4:** Four non-functional regression models with different predictors, range of angle (RoM) and angular velocity (RoV), two functional models considering the curves of angle $\varphi (t)$ and angular velocity $\omega (t)$, and two functional models with scalar predictors. The notation used is the same as that of Table 1. The table also shows the values of multiple correlation *r* and coefficient of determination $R^2$ of the model, as well as the statistic *F* and the *p*-value.

Type	Model	*r*	$R^2$	*F*	*p* value
Non-functional	$ndi \sim RoM$	0.017	<0.001	0.015	0.902
	$ndi \sim RoV$	0.298	0.089	5.174	0.027
	$ndi \sim RoM + RoV$	0.349	0.122	3.597	0.034
	$ndi \sim RoM + RoV + RoM:RoV$	0.352	0.124	2.401	0.078
Functional	$ndi_i \sim \varphi _i(t)$	0.563	0.317	2.322	0.031
	$ndi_i \sim \omega _i(t)$	0.640	0.410	3.475	0.002
Functional plus scalars	$ndi_i \sim \omega _i(t) + RoV$	0.640	0.410	3.060	0.005
	$ndi_i \sim \omega _i(t) + RoV + RoM$	0.643	0.413	2.755	0.009

**Table 5:** Values of statistic *F* and *p* value to compare the nested models to assess whether adding predictors to the simpler model $M_0$ improves it. Two cases of nested models are compared, the first in which scalar variables are added to the functional velocity model $M_{01}$. The second one is when the functional velocity is added to the non-functional model $M_{02}$. The notation used in the model is the same used in Table 4.

Nested models	Model	*F*	*p* value
$M_{01}$: Functional	$ndi_i \sim \omega _i(t)$	0.124	0.884
$M_1$: Functional and scalar predictors	$ndi_i \sim \omega _i(t) + RoV + RoM$		
$M_{02}$: Scalar	$ndi \sim RoM + RoV$	2.378	0.028
$M_1$: Functional and scalar predictors	$ndi_i \sim \omega _i(t) + RoV + RoM$		

### Interpretation of the coefficient function

In addition to improving the model’s predictive ability, functional regression can help interpret the relationship between the predictor curve and the scalar response. To do this, we will describe the contribution of each part of the curve $\omega (t)$ to the value of the NDI based on the value of the product $\beta_1 (t) \omega (t)$ throughout the movement cycle.

**Figure 2:**
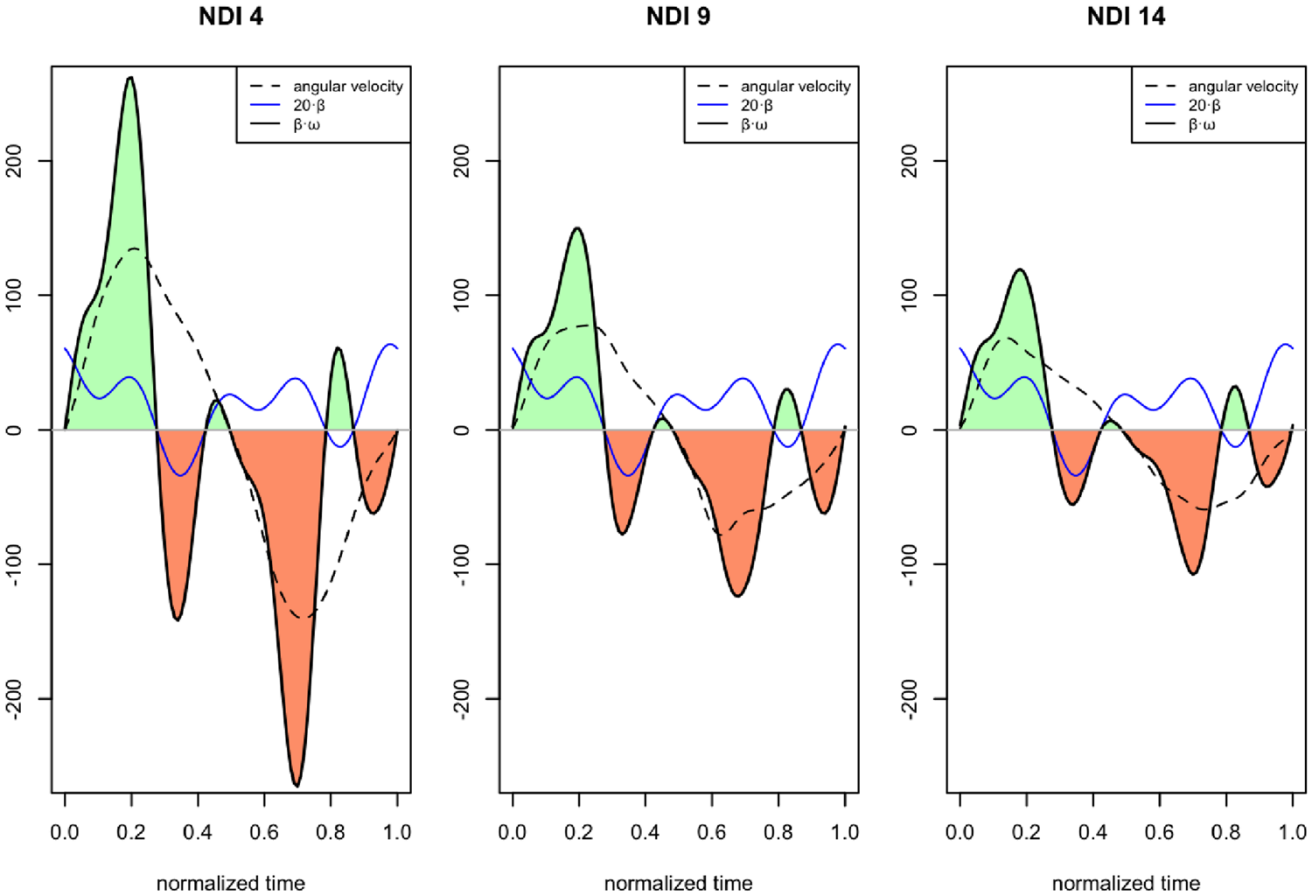
Results for subjects with different levels of NDI: **left**, $NDI= 4$; **middle** $NDI= 9$ and **right** $NDI=14$. The dashed black line is the velocity with the corresponding NDI, the solid blue line corresponds to the scaled coefficient function, and the solid black line corresponds to the product of the velocity and the coefficients function. It is also filled in green (positive area) and red (negative), the regions corresponding to the product function obtained by multiplying the coefficients and velocity functions. The three subjects shown in the figure were selected by choosing three values of low, medium, and high NDI according to the available data whose residual is low, i.e., the predicted NDI is similar to the observed one.

Figure 2 shows the curves corresponding to three cases with different NDIs (4, 9, and 14, respectively). Each graph shows the angular velocity curve $\omega (t)$, dashed black, the adjustment coefficient curve $\beta_1 (t)$ in blue, and the product curve $\beta_1 (t) \omega (t)$ in black and solid.

According to the functional setting, the value of NDI will be estimated from a constant $\beta _0=14.95$ plus the integral $\int \beta _1(t) \omega (t) dt$, which is given by the areas of green regions (positive values) and orange regions (negative values) in the examples in Fig. 2. As can be seen in this figure, in the three cases analyzed, there are three areas with a positive contribution and three with a negative contribution. Although we will make the description based on these three examples, the pattern is similar in all the records. If the areas marked green are higher than the orange ones, the predicted NDI will be greater than the average. Otherwise, it will be lower.

The main contribution to the increase in NDI is in the positive zone from the beginning of the cycle (maximum extension, until reaching the maximum extension velocity). Then, two other zones contribute to a much lesser extent, one around the middle of the cycle (maximum extension) and another small peak after passing through the neutral position after maximum flexion velocity. The zones of negative area and contribution to the decrease in the predicted NDI are also three and appear after the peak of extension velocity, during the acceleration phase until after reaching the maximum at the end.

**Figure 3:**
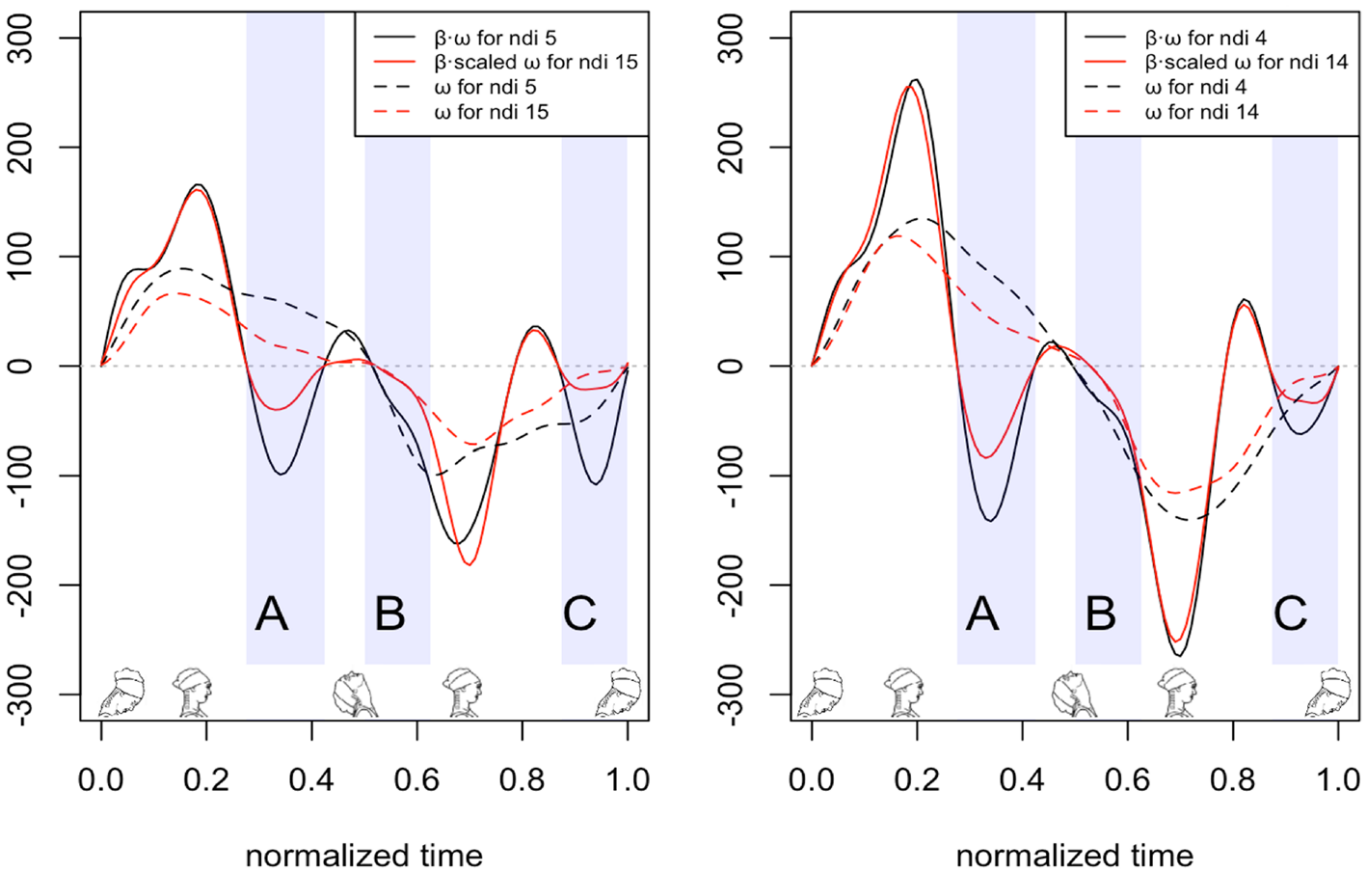
Scaled products functions of beta and velocity function for two subjects with different NDI but similar velocity range. The plotted function is scaled by the constant obtained dividing the maximum values of velocity of the subject with low and high NDI. Velocity functions of each subject are not scaled. Schematic drawings of head positions show as a guide the phase of the corresponding movement, however it is different between subjects due to the normalized time.

RoV is related to the maximum velocities in flexion and extension movements, which are correlated variables. Furthermore, their contributions to the estimated NDI are opposite. This is why the correlation between RoV and NDI is so limited. In fact, it is relatively common to find subjects with similar RoV values but with differences in the NDI, because they present different shapes of the curves. In Fig. 3, two pairs of subjects with these characteristics appear. We will use these examples to illustrate better the contribution of each part of the curve to the increase or decrease in NDI.

The black lines correspond to the subject with the lowest NDI (5 in the left and 4 in the right case), and the red lines correspond to the subject with the highest NDI (14 and 15, respectively). The dashed lines are the velocities of each subject. In solid lines are the product function $\beta_1 (t) \omega (t)$, scaled for greater NDI with the RoV ratio. Three movement zones designated with A, B, and C have been shaded to better comment on the results. Schematic figures of head movement have also been provided to help understand which movement phase corresponds to each zone.

As shown in Fig. 3, in the two graphs, the maximum and minimum of the scaled product function take similar values in each pair of subjects, although they have a very different NDI. Therefore, the contribution of the RoV to the differences in the NDI cannot be substantial. The differences between the $\beta_1 (t) \omega (t)$ curves are observed above all in the shaded areas A, B, and C. Areas A and C correspond to the braking phase of the extension and flexion movements, respectively. Here, the peak for subjects with a lower NDI is more prominent, so there is a larger area under the curve, and therefore, the total NDI value decreases. The shaded area B corresponds at the beginning of the flexion, and the subjects with a lower NDI have higher angular velocity than those with a high NDI, so they have a larger area in this area. These negative areas would explain the differences in the NDI more than the extremes of angular velocities.

## Discussion

The analysis of cervical kinematics is helpful in evaluating the functional alterations associated with neck pathologies. Numerous studies associate pain and injury severity with decreased ROM and slower movements^19^.

However, surprisingly, the correlation between kinematics and the most commonly used assessment scales to quantify the level of pain or functional losses, such as the DASH, NDI, and other scales, is generally low, with values less than 0.4^11,12,14,19,21^. Given that the aforementioned scales have good clinimetric characteristics and are widely used in clinical practice^9^, it is worth asking what the reasons for this weak relationship are, which calls into question the clinical usefulness of kinematic techniques.

One of the possible causes is the way of representing the information contained in the movement curves, which is usually limited to extreme values or ranges, representing a loss of information. Furthermore, it is possible that the functional alterations are not related only to the extreme values but to other characteristics of the movement curves, which are lost when choosing only these extremes. This paper proposes a functional approach to establish the relationship between the kinematic variables (position and speed curves) and the functional state quantified using the NDI scale in a sample of patients with non-specific neck pain and moderate and low levels of disability.

Functional models involve a dimensionality problem since a continuous curve can contain hundreds of observations associated with sampling time instants. This problem is solved by representing the curves using functional basis. In our example, Fourier bases limit the dimensionality to the coefficients of a few functions without losing information from the original curves. We have developed a procedure to identify the optimal number of functions to preserve all the curve information, achieve a good model fit, and ensure its robustness.

The results of different regression models using numerical variables (RoM, RoV, RoM+RoV) have been compared with functional models based on angle, angular velocity and mixed numerical and functional models.

The results show that the functional models have a much closer relationship with the NDI scale than the corresponding ones based on numerical variables. Regarding numerical variables, the model based on the velocity range is better than the one using the RoM ($r=0.298$ vs. $r=0.017$). These results are consistent with other studies, where movement velocity appears more related to disability or pain than range^20,22,23^. However, the correlations with the NDI are much higher when the functional variables replace the ranges. Thus, based on the velocity curve, the simplest functional model presents a correlation with the NDI of $r= 0.640$, more than double the model.

Furthermore, the fit is noticeably better, as shown by the *F* value and the significance level. On the other hand, the comparison of the nested models confirms that the functional model based on the velocity curve is significantly better than any models based on ranges. Thus, adding the range variables to the functional model does not provide a significant improvement, but adding the velocity curve to a range model represents a substantial improvement in the fit (Table 5).

This result confirms that the maximum and ranges of motion or velocity are not necessarily the best parameters to represent the level of mobility or to establish relationships with disability. Considering the entire curve, more information is preserved, strengthening the relationship. Our values are higher than those obtained in other studies^11,12,14,19,21^ in which patients with a much greater range in NDI values have been analyzed.

On the other hand, the functional approach offers a way to interpret which parts of the movement curve are most related to the variation in the NDI index. Indeed, although, in general terms, higher NDI values are associated with lower values of peak flexion and extension velocity, this weak general trend does not explain why patients with similar RoV values have very different NDI values. On the other hand, such differences can be interpreted from a functional point of view. Thus, as mentioned in the results section, the areas of the $\beta_1 (t) \omega (t)$ curve that establish the differences appear immediately after the peak of extension velocity (zone A in Fig. 3) in the starting zone of the flexion movement (zone B in Fig. 3) and the braking at the end of the flexion cycle (zone C). Subjects with higher NDI have lower angular velocity after the peak of extension velocity (zone A, Fig. 3), start with lower angular velocity in the flexion movement (zone B, Fig. 3), and stop earlier at the end of the flexion movement (zone C). In the functional model, it is observed that the contribution of the maximum extension and flexion speeds contribute oppositely to the increase in NDI, which could explain the poor correlation between RoV and NDI.

Scalar-on-function regression has been applied in medical applications^42,50^, but, as far as we know, it is the first work where a functional approach is used to analyze the relationship between biomechanical variables and incidences of disability. The planted model can be found in different R packages and does not present any complexity or computational cost.

In this work, a simple movement has been analyzed, neck flexion, and in a specific pathology, nonspecific neck pain. The sample of patients surveyed corresponds to subjects with low NDI values, so it is expected that the results will be better in others with a broader range of severity levels, as they are subjects with more variability in the kinematic variables and the NDI.

It is difficult to compare our results with those obtained in other studies due to the dispersion in the types of movements, pathologies analyzed, and levels of disability. Since we have not found any work using functional regression, we will limit the comparison of our numerical correlation coefficients between the NDI and the RoM or the RoV with those obtained in other studies where continuous movements have been analyzed to obtain this correlation.

Regarding the NDI-RoM correlation, our result ($r=0.017$) is similar, although slightly lower, to those of other studies where patients with chronic pain and similar levels of disability have been studied. For example, in another study^19^ an $r=0.105$ was obtained, and in other^22^ $r=0.11$. Other studies have shown stronger correlations, $r=0.243$^20^ and ($r=0.250$)^21^. However, these studies focused on patients with other pathologies (neck cancer and migraines, respectively) and with a much wider range of NDI levels. In any case, the improvement in the correlation when applying functional regression is evident compared to that obtained with numerical variables in any of these studies.

Regarding the NDI-RoV correlation, our results align with those cited previously^19,21,22^ with *r* values of 0.30 or lower. Those reported in other study^20^ are somewhat higher ($r=0.4$ in slow movements), but it should be noted that this work analyzes patients with pathology and levels of disability very different from ours. The improvement when applying the functional method represents more than doubling the *r* with the RoV and is much higher than any of the *r* obtained in the studies cited.

In all the papers consulted, the correlation between the NDI and the RoV is higher than between the NDI and the RoM. This result also appears in a more detailed study with partial correlations^23^ and it is consistent with our results from the numerical and functional approaches.

Finally, it should be noted that in the studies cited, continuous records of continuous movement are used, but only to obtain numerical variables corresponding to the extreme values, which, as shown in the results of our study, are not the characteristic most related to the disability levels measured with the NDI. On the contrary, the functional approach allows us to identify which characteristics of the movement curves are associated with differences in the NDI.

This study is groundbreaking in its use of all movement curve data to assess the degree of disability caused by pain, as indicated by the NDI. While the results are promising, there are some limitations to consider. Firstly, the study only evaluated one of the three planes of neck motion - the flexion-extension plane - as it is one of the most commonly used movements in daily life activities (DLA) and has a greater impact on these activities. However, future studies should also address cervical rotation movements, which are also common in DLA, as well as lateralization, which is often coupled with rotation. Additionally, the sample size could be expanded by specifying subgroups based on the type of pathology. Currently, the study includes various pathologies that share the same symptomatology of pain. By characterizing lesions or levels of affectation of a given pathology, this approach could be used to improve understanding of the specific types of pain experienced by patients.

Although the results of this preliminary study seem promising, it is necessary to verify whether the advantages described can be extrapolated to the study of other movements of clinical interest, such as gait analysis, where, in addition, movement patterns with more complex information appear than those analyzed here. In any case, it is logical to expect that the use of complete movement curves, which already contain in their information the maxima, ranges, and other numerical variables used in kinematic analyses, will contribute to improving the goodness of fits between kinematic variables and the assessment scales used in clinical practice.

## Data Availability

The datasets used during the current study are available from the corresponding author on reasonable request.
